# A large-scaled corpus for assessing text readability

**DOI:** 10.3758/s13428-022-01802-x

**Published:** 2022-03-16

**Authors:** Scott Crossley, Aron Heintz, Joon Suh Choi, Jordan Batchelor, Mehrnoush Karimi, Agnes Malatinszky

**Affiliations:** 1 Georgia State University, Atlanta, GA USA; 2 CommonLit, Baltimore, MD USA

**Keywords:** Readability, Corpus linguistics, Readability formulas, Natural language processing

## Abstract

This paper introduces the CommonLit Ease of Readability (CLEAR) corpus, which provides unique readability scores for ~ 5000 text excerpts along with information about the excerpt’s year of publishing, genre, and other metadata. The CLEAR corpus will provide researchers interested in discourse processing and reading with a resource from which to develop and test readability metrics and to model text readability. The CLEAR corpus includes a number of improvements in comparison to previous readability corpora including size, breadth of the excerpts available, which cover over 250 years of writing in two different genres, and unique readability criterion provided for each text based on teachers’ ratings of text difficulty for student readers. This paper discusses the development of the corpus and presents reliability metrics for the human ratings of readability.

Reading is an essential skill for academic success. It is important to support and scaffold literacy challenges faced by students by selecting texts of difficulties appropriate for their reading abilities. Providing students with texts that are accessible and well matched to their abilities helps to ensure that students better understand the text and, over time, can help readers improve their reading skills (Mesmer, 2008; Stanovich, 1985). Readability formulas, which provide an overview of text difficulty, have shown promise in more accurately benchmarking students with their text difficulty level, allowing students to read texts at target readability levels.

Most educational texts are calibrated using traditional readability formulas like Flesch–Kincaid Grade Level (FKGL; Kincaid et al., 1975) or commercially available formulas such as Lexile (Smith et al., 1989) or the Advantage-TASA Open Standard (ATOS; School Renaissance Inst. Inc, 2000). However, both types of readability formulas are problematic. Traditional readability formulas lack construct and theoretical validity because they are based on weak proxies of word decoding (i.e., characters or syllables per word) and syntactic complexity (i.e., number or words per sentence) and ignore many text features that are important components of reading models including text cohesion and semantics. Additionally, many traditional readability formulas were normed using readers from specific age groups on small corpora of texts taken from specific domains. Commercially available readability formulas are not publicly available, may not have been tested rigorously for their reliability, and may be cost-prohibitive for many schools and districts, let alone teachers.

In this paper, we introduce the open-source CommonLit Ease of Readability (CLEAR) corpus. The corpus is a collaboration between CommonLit, a non-profit education technology organization focused on improving reading, writing, communication, and problem-solving skills, and Georgia State University (GSU), with the end goal of promoting the development of more advanced and open-source readability formulas that government, state, and local agencies can use in testing, materials selection, material creation, and other applications commonly reserved for readability formulas. The formulas that will be derived from the CLEAR corpus will be open-source and ostensibly based on more advanced natural language processing (NLP) features that better reflect our understanding of the reading process. The accessibility of these formulas and their reliability should lead to greater uptake by students, teachers, parents, researchers, and others, increasing opportunities for meaningful and deliberate reading experiences. We outline the importance of text readability along with concerns about previous readability formulas below. In addition, we present two studies that examine the reliability of the CLEAR corpus by discussing the methods used to develop the corpus, examining how well traditional and newer readability formulas correlate with the reading criteria reported in the CLEAR corpus, and developing a new readability formula to assess how individual features in CLEAR excerpts are predictive of CLEAR reading criteria.

## Text readability

Text readability can be defined as the ease with which a text can be read (i.e., processed) and understood in terms of the linguistic features found in that text (Dale & Chall, 1948; Richards et al., 1992). However, in practice, most research into text readability is more focused on measuring text understanding (i.e., comprehension Kate et al., 2010) and not the speed at which a text is read (i.e., text processing). Text comprehension is generally associated with the contents of the text including word sophistication, syntactic complexity, and discourse structures (Just & Carpenter, 1980; Snow, 2002), all of which relate to text complexity. Text comprehension is also a function of a reader’s reading proficiency and background knowledge of the text (McNamara et al., 1996). However, for the purpose of this study, we will focus only on text features.

Many studies have revealed that word sophistication features such as sound and spelling relationships between words (Juel & Solso, 1981; Mesmer, 2005), word familiarity and frequency (Howes & Solomon, 1951), and word imageability and concreteness (Richardson, 1975) can result in faster word processing and more accurate word decoding. The meaning of words, or semanticity, also plays an important role in text readability, in that readers must be able to recognize words and know their meaning (Mesmer et al., 2012). Therefore, word semanticity and larger text segments can facilitate the linking of common themes and easier processing based on background knowledge and text familiarity (Bailin & Grafstein, 2001; McNamara & Kintsch, 1996).

Effective readers are also able to parse syntactic structures within a text to help organize main ideas and assign thematic roles where necessary (Graesser et al., 1996; Mesmer et al., 2012). Two features that allow for quicker syntactic parsing are words or morphemes per t-unit (Cunningham et al., 2005) and sentence length (Klare, 1984). Parsing information in the text helps readers develop larger discourse structures that result in a discourse thread (Grimes, 1975). These structures, which relate to text cohesion, can be partially constructed using linguistic features that link words and concepts within and across syntactic structures (Givón, 1995). Sensitivity to these cohesion structures allows readers to build relationships between words, sentences, and paragraphs, aiding in the construction of knowledge representations (Britton & Gülgöz, 1991; Kintsch, 1988; McNamara & Kintsch, 1996). Moreover, such sensitivity can help readers understand larger discourse segments in texts (Gernsbacher, 1990; Mesmer et al., 2012).

Additionally, the genre of a text may influence ease of readability. Studies have demonstrated that narrative texts are generally more comprehensible than informative or expository texts (Best et al., 2008; Sáenz & Fuchs, 2002). Importantly, narrativity can be cued through text features. For instance, Biber (1988) found that text features based on part of speech tags could distinguish between narrative and non-narrative texts. Specifically, he reported that narrative texts included more instances of past tense verbs, third person pronouns, perfect aspect, public verbs, negation, and present participial clauses. More recently, Graesser et al. (2011) used a principal component analysis to extract readability components from the Touchstone Applied Science Associates (TASA) corpus, which comprises over 35,000 excerpts from language arts, science, and history texts. They used the NLP tool Coh-Metrix (Graesser et al., 2004) to extract linguistic features from the excerpts and found that the strongest component represented narrativity. The narrativity component was comprised of text features related to the occurrence of verbs and pronouns.

## Traditional readability formula

In line with findings from discourse processing studies, several traditional readability formulas include word sophistication and syntactic complexity features (e.g., Chall & Dale, 1995; Flesch, 1948; Kincaid et al., 1975). However, traditional readability formulas tend to disregard semantic features, narrativity aspects, and discourse structures found in texts. Moreover, traditional readability formulas rely on proxy estimates for lexical and syntactic features. For example, number of characters per word is used as a proxy for word sophistication, and number of words per sentence is used as a proxy for syntactic complexity in both the Flesch Reading Ease (Flesch, 1948) and the Flesch–Kincaid Grade Level formulas (Kincaid et al., 1975). The New Dale Chall formula (Chall & Dale, 1995) includes the number of uncommon words in a text as compared to the length of words, but still relies on sentence length to assess syntactic complexity.

The use of proxy measures for text features important in text processing is a pressing criticism of traditional readability formulas, but it is not the only criticism. Traditional readability formulas often lack strong construct validity since the features that they employ are solely based on statistical correlations to develop predictive power and may not be theoretically oriented. A theoretically important component lacking from traditional readability formulas is text cohesion, or the relationship between elements in a text. Text cohesion is an important feature of text comprehension because it helps readers build new knowledge and is an indicator of text readability (Britton & Gülgöz, 1991; Kintsch, 1988; McNamara & Kintsch, 1996). Second, traditional formulas often ignore concerns of style, vocabulary, and grammar, which may equally play a role in text readability (Bailin & Grafstein, 2001). Finally, the reading criteria used to develop traditional formulas are often based on multiple-choice questions and cloze tests, two methods that may not measure text comprehension accurately (Carlisle & Rice, 2004; Magliano et al., 2007).

Above all, the generalizability of most traditional readability formulas is suspect because they have been normed using readers from specific age groups and using small corpora of texts from specific domains. The Flesch Reading Ease formula (Flesch, 1948) and the Dale–Chall formula (Dale & Chall, 1948) are two examples of this concern. These formulas were normed using 350 texts written in the 1920s for 3rd through 12th grade students (the McCall-Crabbs’ Standard test lessons in reading). Another example is the Flesch Kincaid Grade Level formula, which was developed from only 18 passages taken from Navy training manuals, and its readability criterion was collected from roughly 600 navel enlistees. The continued popularity of these formulas today illustrates how these formulas have been generalized and applied to a much wider population than for which they were developed.

## NLP informed readability formulas

A number of more recently developed readability formulas based on more advanced NLP features have been shown to outperform traditional readability formulas (Crossley et al., 2008; Crossley et al., 2019a, b; Feng et al., 2010; Foltz et al., 1998; Pitler & Nenkova, 2008; Sheehan et al., 2014) at predicting text readability. NLP informed readability formulas are both commercially available and open-sourced (e.g., freely available). Generally, advanced NLP readability formulas rely on extracting advanced text features that go beyond length of words and sentences as found in traditional readability formulas.

There are a number of commercially available readability tools including Lexile framework (Smith et al., 1989), the Advantage-TASA Open Standard for Readability (ATOS) formula (School Renaissance Inst. Inc, 2000), the Pearson Reading Maturity Metric (Landauer & Way, 2012) and the TextEvaluator Tool (Sheehan et al., 2014) that incorporate more advanced NLP calculations to assess readability. However, in most cases, the features that underlie these algorithms are proprietary and not openly available to replicate or test. A good example of this is the TextEvaluator tool. The tool, as reported in Sheehan et al. (2014), contains 43 different linguistic features that are aggregated into language components related to readability, including word familiarity, word concreteness, academic vocabulary, syntactic complexity, lexical cohesion, narrativity, interactive/conversational style, and argumentation. However, information on the databases and the algorithms that underlie these features are not available.

In contrast, there are a number of open-source readability formulas that report on specific linguistic features used to inform readability formulas. A few of these formulas focused on syntax by using a parser to calculate features such as the incidence of verb and noun phrases, parsing tree depth, and embedded clauses in a text (Feng, Jansche, Huenerfauth, & Elhadad; Schwarm & Ostendorf, 2005). Other formulas focus on word complexity (Collins-Thompson & Callan, 2005) or the frequency of grammatical constructions (Heilman et al., 2006). Still, other formulas combine measures of syntactic complexity, lexical sophistication, and cohesion (Crossley et al., 2007; Crossley et al., 2008; Crossley et al., 2019a, b; Newbold & Gillam, 2010; Pitler & Nenkova, 2008). Newer readability formulas are based on neural network approaches using transformer models (Martinc et al., 2021).

In general, readability formulas based on more advanced NLP features are reliable and outperform traditional readability formulas. In a large study of commercially available formulas, Nelson et al. (2012) found that NLP informed readability formulas were reliable and, often, highly correlated with student reading performance or differences in grade levels across a variety of texts. Similarly, Sheehan et al. (2014) found that the NLP features reported by TextEvaluator were highly correlated with grade level judgment of text complexity, were reliable across informational and literary texts, and avoided blueprint bias (i.e., bias based on readability formulas being trained on texts from a single reading assessment). Open-source formulas have also been shown to be reliable as well as outperform traditional readability formulas. For example, formulas reported in Collins-Thompson and Callan (2005) and Crossley et al. (2007) reported increased text classification accuracy using NLP features, and Crossley et al. (2008), Feng et al. (2010), Newbold and Gillam (2010), and Pitler and Nenkova (2008) found that NLP informed readability formulas outperformed traditional readability formulas. Additionally, François and Miltsakaki (2012) report that while classic linguistic found in traditional readability formulas are strong predictors of readability, they are not better predictors than more advanced NLP features and that leaving out advanced NLP features can negatively impact performance. However, Francois and Miltsakaki reported the strongest performance for readability models that included both classic and advanced NLP features.

## Current research

This paper introduces the CommonLit Ease of Readability (CLEAR) corpus and assesses its reliability in two studies. The first study provides background on the curation of the corpus, the human ratings of the text excerpts (i.e., the CLEAR scores), and analyses of these ratings using corpus information. Specifically, we discuss methods used to bolster the internal reliability of the corpus and analyses that examine scoring reliability by investigating differences in the human scores based on year of publication and genre (informative versus literary texts). Our second study examines reliability of the human scores based on analyses of text features found in the excerpts to examine how predictive text features known to be related to comprehension are of the human ratings. In the second study, we examine overlap between the human scores and traditional and newer readability formulas. In a follow-up study, we develop a readability formula based on the human ratings and the language features in the individual texts as measured by NLP techniques to examine how individual text features are predictive of human scores.

## Study 1: Corpus collection, scoring, and internal reliability

Our first study focuses on the curation of the excerpts used in the corpus including initial selection and criteria for final inclusion. We also discuss the scoring procedure used to provide individual scores of text readability for each excerpt in the corpus. Lastly, we report on assessments of internal reliability within the corpus using available metadata provided about the corpus.

## Method

### Initial corpus collection

We collected text excerpts from CommonLit’s reading sample database, Project Gutenberg, Wikipedia, and dozens of other open digital libraries. Excerpts were manually selected from the beginning, middle, and end of texts and only one sample was selected per text. Text excerpts were selected to be between 140 and 200 words, with all excerpts beginning and ending at an idea unit (i.e., excerpts were manually selected so that they did not end in the middle of sentences or ideas). The text excerpts were written between 1791 and 2020. We selected this wide range of texts to both represent texts found in the classroom and language over time. The majority of excerpts were selected between 1875 and 1922, when copyrights expired affording free use of the data, and between 2000 and 2020, when non-copyright texts became available on the Internet. Of the 4724 passages in the corpus, 3194 are public domain, 1253 used various Creative Commons licenses, and 277 used GNU or mixed-source licenses. The most popular single license, "CC BY 4.0" was used by 817 excerpts.

Excerpts were selected from two genres: informational and literature texts. Excerpt classification followed industry-standard practices based on the Partnership for Assessment of Readiness for College and Careers (PARCC) passage selection guidelines. These guidelines classify informational texts as non-fiction, history/social science, science/technical, and digital texts. Poetry, drama, fiction, and multi-media texts are considered literary. We started with an initial sample of ~ 7600 texts. Each excerpt was read by at least two material developers employed by CommonLit to judge the text’s acceptability. The two major criteria for acceptability were the likelihood of being used in a 3rd–12th grade classroom and whether or not the topic was appropriate using Motion Picture Association of America (MPAA) ratings (e.g., G, PG, PG-13). Each text was read by at least two raters and evaluated on likelihood of use and MPAA ratings. Excerpts that were flagged as potentially inappropriate were then read by a third expert rater and either included or excluded from the corpus. We also conducted automated searches for traumatic terms (e.g., terms related to racism, genocide, or sexual assault). Any excerpt flagged for traumatic terms was also reviewed by an expert rater. Author representation was limited such that a single author could contribute no more than 12 excerpts within the corpus. After removing excerpts based on these criteria, we were left with 4793 excerpts. These excerpts were copy-edited to ensure that they did not contain grammatical, syntactic, and spelling errors. Line-breaks were standardized across the excerpts. Lastly, selected archaic spellings (e.g., to-day, Servia) were replaced with modern spellings (e.g., today, Serbia).

### Human raters

We recruited ~ 1800 teachers from the CommonLit teacher pool through an e-mail marketing campaign. Teachers were asked to participate in an online collection experiment. First, the teachers completed a background survey that asked for their gender, age, education level, and first language. The survey also asked for their confidence and enjoyment in reading and writing (on a 1–6 scale). Teachers were then expected to read 100 pairs of excerpts and make a judgment for each pair as to which excerpt was easier to understand. Teachers were paid $50 in an Amazon gift card for their participation.

**Fig. 1:**
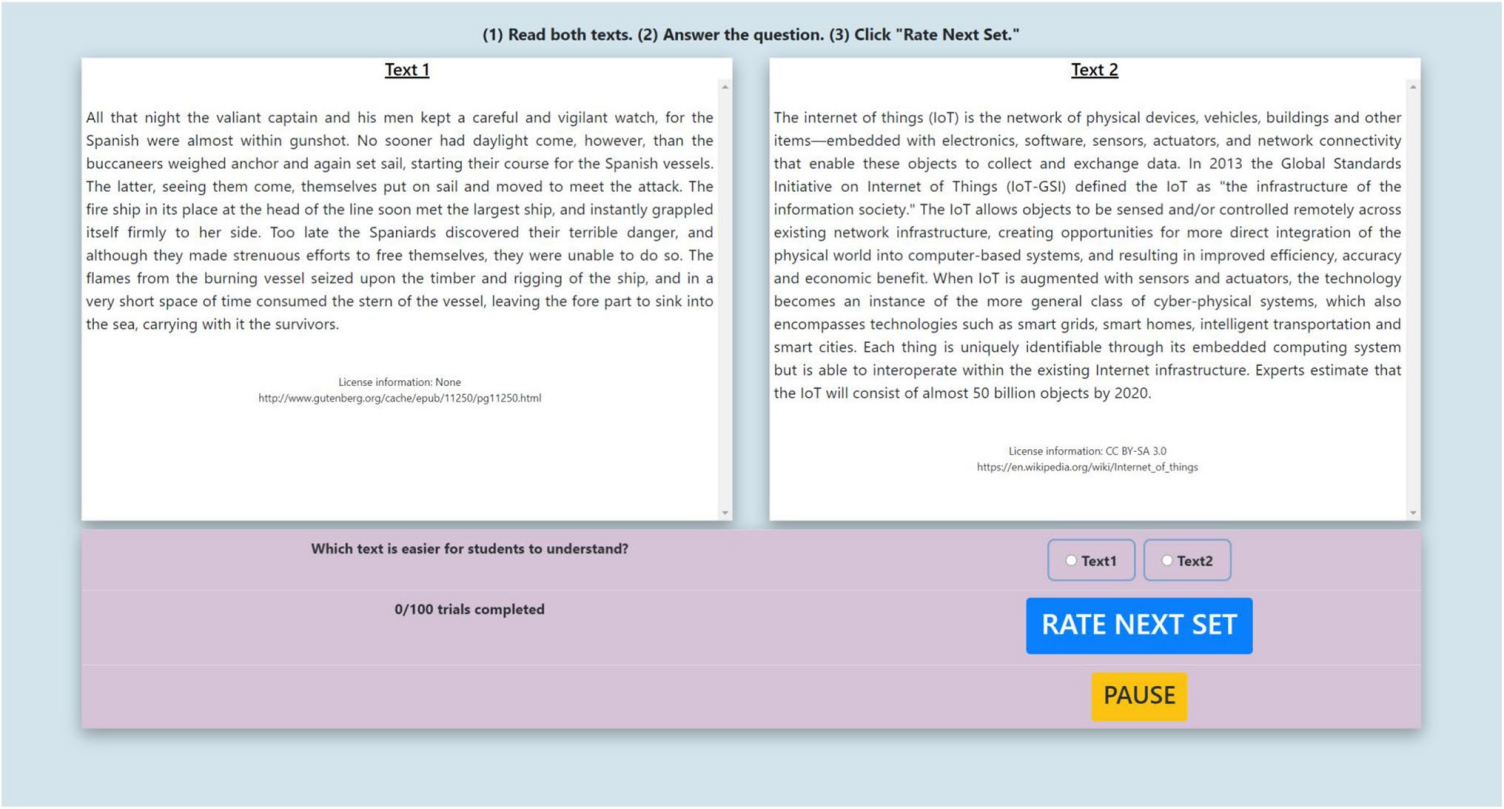
Rater interface for pairwise readability comparisons

For data collection, we developed an online website. The basic format for the readability judgments was to show two excerpts side by side and ask participants to judge which of the two texts would be easier for a student to understand using a checkbox format. There were two additional buttons on the website. The first moved the participant to the next comparison and the second allowed participants to pause the experiment. The website also included a progress tally to show participants how many comparisons they had made (see Fig. 1 for screenshot of pairwise comparison task). We asked teachers to conduct text comparisons because such approaches have been successfully used in the past (Crossley et al., 2017a, b; Crossley et al., 2019a, b; De Clercq et al., 2014). Additionally, collecting text comparisons is less resource intensive than collecting reading criteria through direct assessment (e.g., cloze tests, multiple choice tests, text summarizations, Crossley et al., 2019a, b).

The website first provided participants with informed consent and an overview of the expectations. The website then collected survey information. Participants were then given a practice excerpt comparison to familiarize them with the design. After the practice comparison, participants moved forward with the data collection. Excerpts were paired randomly, and excerpts were shown on either the right or left-side panel randomly. The licensing information and the uniform resource locator (URL) for each text were displayed on the bottom side of each panel. Participants were redirected to a break screen after completing every 20 comparisons. The break screen showed how much time (in total and per comparison) the participant had spent on the task. A button allowing the participant to continue to the next comparison appeared after spending one minute on the break screen, meaning that the participants were required to take at least a one-minute break per 20 comparisons. After completing 100 comparisons, the participants were given a completion code that they could redeem for the gift card. The website was written in Python, JavaScript, CSS, and HTML. The website was housed on a cloud server.

### Rater reliability

Of the ~ 1800 participants that initially logged into the experiment, 1198 completed the entire experiment. However, not all participant data was kept. We removed participants who did not complete the entire experiment. We also removed participants to increase the reliability of the pairwise scores based on deviant patterns and time spent on judgments. In terms of deviant patterns, we removed all participants who selected excerpts in either the right or left panel more than 70% of the time. We also removed participants who had binary patterns of selecting left/right or right/left panels more than 20 times in a row. In terms of time spent on judgments, we removed participants who spent less than 10 s on average per comparison and/or spent a median time under 5 s. After removing participants based on patterns and time, we were left with data from 1116 participants.

### Final participant data

The final 1116 participants made 111,347 overall comparison judgments (M = 99.773 judgments per participant). On average, each excerpt was read 46.47 times (SD = 6.63) and participants spent an average of 101.36 s per judgment. However, we did not remove participants for taking too long on judgments, since pauses were allowed. Thus, our data for time was right skewed.

In terms of demographics, the average age of the raters was 40.87 years old (SD = 10.23). The majority of raters were female (*n* = 970) with the remaining identifying as male (*n* = 145) or other (*n* =1). Of the 1116 raters, 771 of them reported having some graduate school education. The rest reported having a bachelor’s degree. Most of the raters were native speakers of English (*n* = 1080) with a small population of non-native speakers (*n* = 36). On average, the raters showed an above average confidence in reading (M = 5.59, SD = 1.03) and writing (M = 5.08, SD = 0.95). The raters also showed higher enjoyment in reading (M = 4.62, SD = 0.64) than writing M = 3.79, SD = 0.90). The participants showed – on a scale of 1 to 6 – high confidence in their writing (5.06) and reading skills (5.54). Most of the participants (over 99.9%) were self-reported native speakers of English. Most of the participants reported – on a scale of 1 to 5 – that they enjoyed reading (4.59) more than they did writing (3.80). The participants reported that they watched around 2.5 hours of TV a day on average.

### Pairwise ranking scores

To calculate unique readability scores for each excerpt, we used a Bradley–Terry model (Bradley & Terry, 1952) to compute pairwise comparison scores for the human judgments of text ease. This is a similar approach to computing readability scores as reported by Crossley, Skalicky, et al. (2017b), Crossley et al. (2019a, b) and De Clercq et al. (2014). A Bradley–Terry model describes the probabilities of the possible outcomes when items are judged against one another in pairs (see Eq. 1). The Bradley–Terry model ranks documents by difficulty based on each excerpt's probability to be easier than other excerpts. The model creates a maximum likelihood estimate which iteratively converges towards a unique maximum that defines the ranking of the excerpts (i.e., the easiest texts have the highest probability).

Bradley–Terry Model1

After computation, the Bradley–Terry model provides a coefficient for each text along with a standard error. These coefficients were continuous variables that ranged from ~ – 3.5 to ~ 1.5. The coefficients were not scaled to grade level. We examined both coefficients and standard errors for outliers. We found 52 texts that had a coefficient with a standard deviation greater than 2.5 and an additional 17 excerpts with a standard error greater than 0.65. These were removed from the final dataset leaving us with a sample size of 4724.

### Final corpus data

The final CLEAR corpus comprised 4724 texts, each with a unique CLEAR score calculated using a Bradley–Terry model. These texts had an average publication year of 1937.89 (SD = 60.51) and were generally split between informative texts (*n* = 2304) and literary texts (*n* = 2420). The texts had an average Flesch–Kincaid Grade Level score of 9.51 (SD = 4.33). The majority of excerpts were taken from the middle of texts (*n* = 3470) followed by the beginning (*n* = 1024) and end (*n* = 108) of the text. A small number of excerpts comprised an entire text (*n* = 122). In terms of content, most of the texts were rated General (G, *n* = 3706) followed by Parental Guidance (PG, *n* = 928), and PG-13 (*n* = 87). Three Restricted (R) rated texts were also kept in the final data set because they were judged to be appropriate for their grade level. Descriptive statistics for the corpus in terms of average number of words, sentences, and paragraphs are provided in Table 1. The corpus along with associated readability scores, demographic information, and other meta-data is available at https://github.com/scrosseye/ CLEAR-Corpus. Descriptions of the columns contained in the dataframe are presented in Table 2.

**Table 1:** Text descriptives for the CLEAR corpus

Variable	Mean	Standard Deviation	Minimum	Maximum
Number of words	178.51	18.46	135	228
Number of sentences	9.57	4.64	2	41
Number paragraphs	2.54	1.87	1	20

**Table 2:** Column descriptions for CLEAR corpus dataframe

Column name	Description
ID	Random ID number
Author	Author of excerpt
Title	Title of excerpt
Anthology	Anthology taken from (if relevant)
URL	Website text retrieved from (if relevant)
Pub Year	Publication year
Categ	Genre of excerpt
Sub Cat	Sub-category of genre (if relevant)
Lexile Band	Lexile Reading Band
Location	Part of text excerpt from which excerpt was extracted
License	License for excerpt (if relevant)
MPAA Max	Maximum MPAA rating given
MPAA #Max	MPAA rating as integer
MPAA# Avg	Average MPAA rating as integer
Excerpt	Text associated with excerpt
Google WC	Word count for excerpt from Google
Sentence Count	Sentence count for excerpt
Paragraphs	Paragraph count for excerpt
BT_easiness	Bradley–Terry ease of readability score
s.e.	Bradley–Terry standard error score
Flesch-Reading-Ease	Flesch-Reading Ease score
Flesch–Kincaid-Grade-Level	Flesch–Kincaid-Grade-Level score
Automated Readability Index	Automated Readability Index score
SMOG Readability	SMOG Readability score
New Dale–Chall Readability Formula	New Dale–Chall Readability Formula score
CAREC	Crowdsourced Algorithm of Reading Comprehension score
CAREC_M	Crowdsourced Algorithm of Reading Comprehension score (controlled for text length)
CML2RI	Coh-Metrix Second Language Reading Index score

### Statistical analyses

To assess the reliability of the CLEAR corpus and the associated ease of readability scores (i.e., CLEAR scores), we conducted multiple different analyses, all conducted in R 4.0.2 (R core team, 2020). We first used split-half reliability to assess the Bradley–Terry scores derived from the full data set. We next examined differences in the CLEAR scores between informational and literary texts using the t.test() function. We then assessed temporal association between the CLEAR scores and year of publishing using Bivariate Pearson correlations reported by the cor.test() function. The R codes used in this study are available at https://github.com/scrosseye/CLEAR-Corpus

## Results

### Split-half reliability

To assess the reliability of the raters and the resulting Bradley–Terry scores for all the data, we used split-half reliability. Split-half reliability is used to measure the consistency of test scores and involves splitting a test into half and correlating scores on the two halves of the tests. In the case of the pairwise comparison data, the participants’ 111,347 comparisons were randomly split into halves. Thus, the first split comprised 55,673 comparisons and the second split comprised 55,674 comparisons. Overall, this meant that for each split, each text was read on average 23 times as compared to 46 times in the full data set. While a lower number of reads should lead to lower reliability, the resulting Bradley–Terry scores for each split should still correlate with scores from the entire rater pool.

**Fig. 2:**
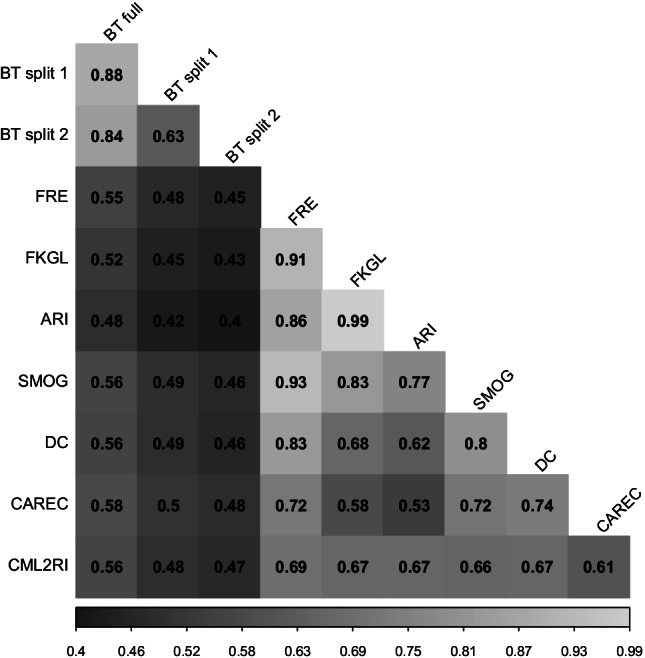
Correlation plot between Bradley-Terry scores (full and split half) and readability formulas

Correlations between the full Bradley–Terry model scores for each text and the Bradley–Terry scores from the two splits are reported in the upper left quadrant of Fig. 2. The results show strong associations between the full model scores and the two splits (*r* = ~.85). The two splits also reported strong correlations with one another, although weaker than with the full model (*r* = .63). The remaining correlations reported in Fig. 2 will be discussed in Study 2 below.

### Genre differences

**Fig. 3:**
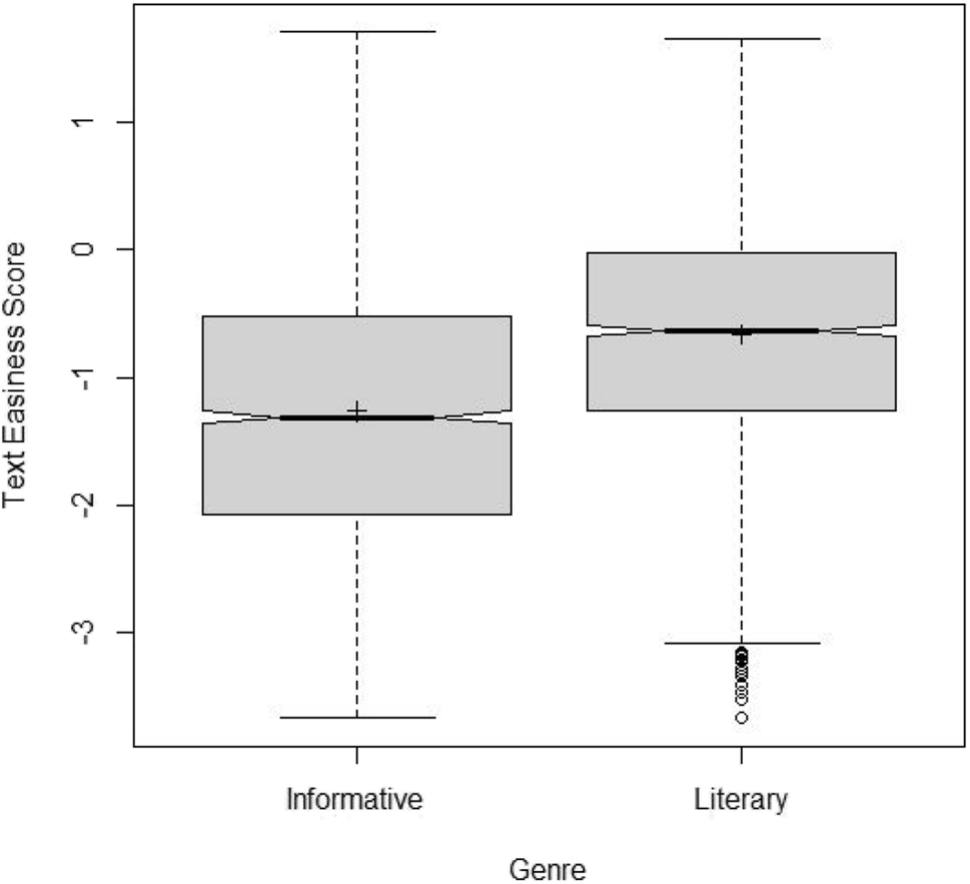
Boxplot for Bradley-Terry scores for excerpts based on genre

We calculated a simple *t* test to examine differences between the reported Bradley–Terry coefficients and the informational and literature categories to examine the hypothesis that informational excerpts (M = – 1.27, SD = 1.06) would be rated as more difficult compared to literature excerpts (M = – 0.663, SD = 0.914). The *t* test supported the hypothesis, (*t*(4723) = – 20.95, *p* < .001), with a medium effect size (*d* = – 0.43, Cohen, 1992). See Fig. 3 for a box plot depicting differences in text categories.

### Temporal associations

**Fig. 4:**
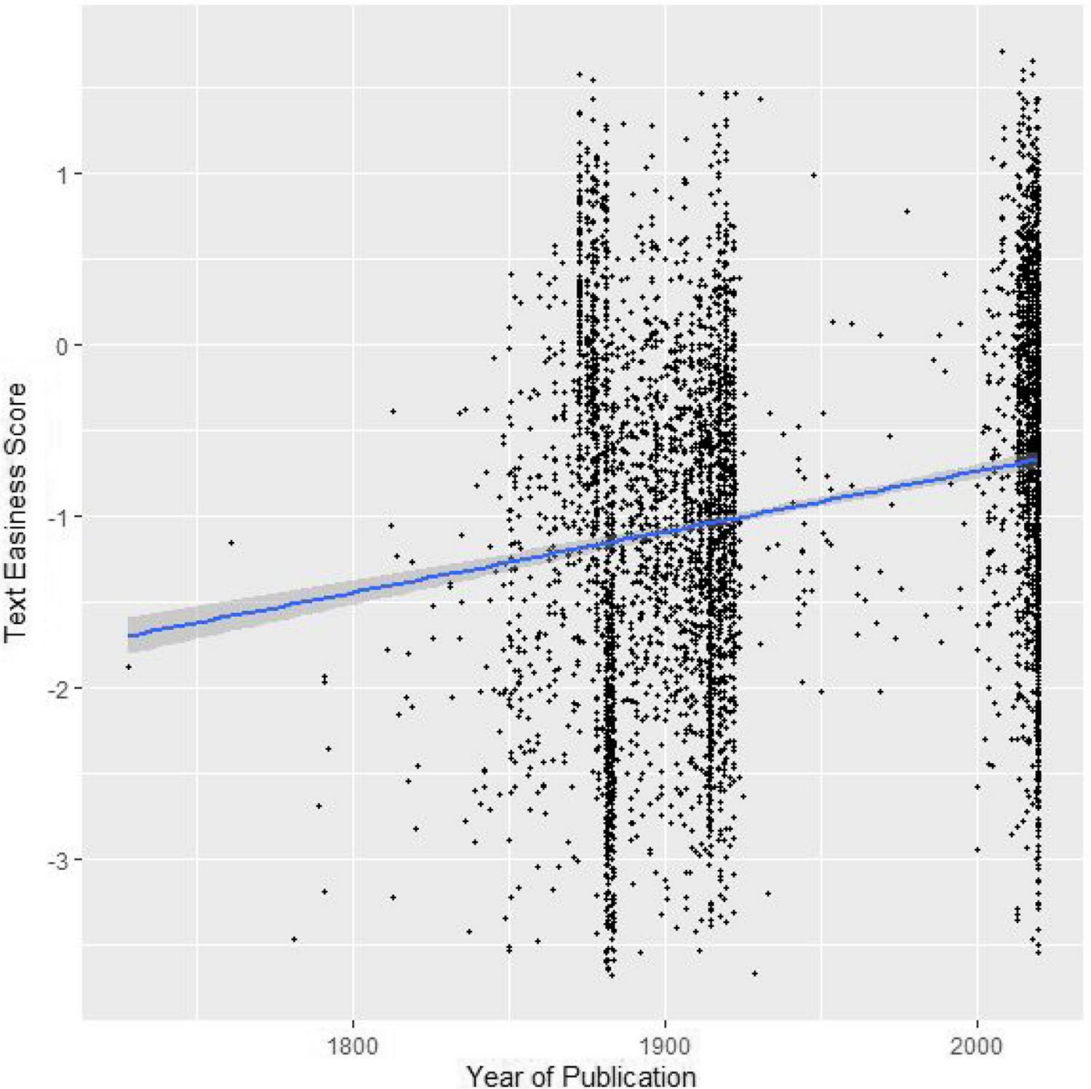
Scatterplot for Bradley-Terry scores by year of excerpt publication

We used a Pearson’s correlation test to test whether Bradley–Terry coefficients were correlated with the excerpts’ year of publication. We were unable to locate publication years for nine excerpts. The correlation reported a weak, but significant correlation (*r*(4715) = .206, *p* < .001) indicating that more recent passages were often rated as easier to read than older passages (see Fig. 4).

## Discussion

The CLEAR corpus was curated using strict measures to ensure that a variety of excerpts leveled for 3rd to 12th graders was selected from both informational and literary genres. All excerpts were selected from open sources to ensure that the corpus follows open science principles. The excerpts were also controlled for text length and varied among location within the larger texts from which they were taken. Importantly, only one excerpt was sampled from each larger text, helping to maintain the independence of the data, which is not the case of all readability corpora (cf., the Touchstone Applied Science Associates [TASA; Zeno et al., 1995] corpus which includes multiple excerpts from the same text). Excerpts were also checked to ensure they contained appropriate topics and the excerpts were thoroughly cleaned and standardized.

Each excerpt in the corpus contains a unique readability score, (i.e., CLEAR score) which allows for the modeling of individual texts. This is in comparison to modeling text based on group characteristics which is common in large reading corpora. For instances, the Newsela corpus (Xu et al., 2015) contains over 5000 texts, but the texts are categorized into five difficulty levels allowing researchers to only develop models of readability based on these levels. Our raters, all trained teachers, were also asked to judge text readability based on student understanding of the texts, helping to ensure that the judgments reflected student text comprehension. Raters with deviant patterns of scoring were removed. In the end, each excerpt was read, on average, over 45 times by 1116 participants allowing us to capture a large amount of population variance.

To assess the reliability of the pairwise scores calculated on the excerpts we examined split-half reliability and differences in scores based on genre and temporality. We found strong associations between the Bradley–Terry scores for the text in the full data set and each split of the dataset providing reliability for the Bradley–Terry scores. Correlations were not perfect, of course, which is to be expected especially considering that Bradley–Terry models are stronger when there are more judgments per items. Because the data splits had half as many judgments as the full split, we would expect lower correlations. For our other analyses, we presumed that literary excerpts would be easier to process than informational excerpts because of their reliance on discourse and narrative structure, and that is what was reported in the statistical analyses. We also presumed that more recent excerpts would be easier to understand than older excerpts because of language change over time, which is also what we found in our analyses.

## Study two: reliability and text complexity

Our second study examines external markers of reliability that focus specifically on readability criteria as a means to predict the CLEAR scores. We do this in two different ways. First, we examine associations between the ease of readability scores and existing readability formulas (e.g., Flesch–Kincaid Grade Level). Second, we develop a new readability formula using existing NLP features to assess how well text features are predictive of the ease of readability scores. Our goal is to demonstrate that the human ratings of reading ease are partially a function of language features in the texts, thus providing reliability to the ratings. If readability formulas and individual text features known to be important in text comprehension are predictive of the human ratings, that we have increased reliability that the ratings are capturing text readability.

### Readability formulas

We calculated a number of readability formulas using the Automatic Readability Tool for English (ARTE, version 1.1; Choi & Crossley, 2020). ARTE provides free and easy access to a wide range of readability formulas and automatically calculates different readability formulas for batches of texts (i.e., thousands of texts can be run at a time) to produce readability scores for individual texts in an accessible spreadsheet output. ARTE was developed to help educators and researchers easily process texts and derive different readability metrics allowing them to compare that output and choose formulas that best fit their purpose. The tool is written in Python and is packaged in a user-friendly GUI that is available for use in Windows and Mac operating systems at linguisticanalysistools.org. The selected readability formulas are discussed in depth below.

- *Flesch reading ease.* Flesch Reading Ease (Flesch, 1948) uses two text metrics – average number of words per sentence and average syllables per word – to produce a score between 0 to 100, with higher scores indicating that a text is easier to comprehend.
- *Flesch–Kincaid grade level.* Flesch Reading Ease was recalculated in 1975 to be more suitable for use in the United States’ Navy (Kincaid et al., 1975). This formula uses the same two metrics as Flesch Reading Ease and produces grade levels for different texts.
- *ARI.* Automated Readability Index (Kincaid et al., 1975) is another readability formula developed under contract to the United States Navy. This formula uses average characters per word instead of average syllables per word to proxy word difficulty.
- *SMOG.* SMOG (McLaughlin, 1969) was inspired by Gunning’s FOG index (Gunning, 1952). The formula uses a single metric to predict text difficulty: the number of polysyllabic words in a text.
- *New Dale–Chall.* The New Dale–Chall (Chall & Dale, 1995) is a recalculation of the original Dale–Chall formula (Dale & Chall, 1948). The New Dale–Chall formula utilizes a list of 3000 words that were classified as being ‘familiar’ by 4th grade students along with average sentence length.
- *CAREC.* The Crowdsourced Algorithm of Reading Comprehension (Crossley et al., 2019a, b) was developed using a corpus of 600 texts across three different genres that had readability criterion based on the judgments of over 800 crowdsourced participants. Unlike the previous formulas, it depends on more advanced NLP tools that calculate features related to syntactic complexity, lexical diversity, and textual cohesion. The formula includes lexical features such as age of acquisition, bigram range, trigram proportion scores, characters per word, imageability, character entropy, and frequency. The formula also includes features related to text cohesion including lexical overlap between paragraphs and the use of temporal connectives.
- *CML2RI*. The Coh-Metrix L2 Readability Index (Crossley et al., 2008) using linguistic features derived from Coh-Metrix (Graesser et al., 2004). The features underlying the formula include word frequency, syntactic similarity between sentences, and content word overlap between adjacent sentences.

### Natural language processing features

To develop a new readability formula to assess the reliability of the human ratings found in the CLEAR corpus, we incorporated NLP features reported by the Suite of Automatic Linguistic Analysis Tools (SALAT; Crossley & Kyle, 2018). We specifically selected tools that report on features related the broad categories of readability reported by Collins-Thompson (2014) including lexical-semantics, syntax, discourse, higher level semantics, and pragmatics. All tools used in this analysis are freely available at linguisticanalysistools.org. The individual tools and their output are discussed below.

### TAALES.

The Tool for the Automatic Analysis of LExical Sophistication (TAALES, version 2.2; Kyle & Crossley, 2015; Kyle et al., 2018) measures text features related to lexico-semantics and higher-level semantics. The inclusion of lexico-semantic and higher level semantic features is based on the notion of the importance of vocabulary in readability and specifically that difficult or more unfamiliar words make a text more difficult to process (Collins-Thompson, 2014).

TAALES calculates lexical features for 135 new and classic lexical sophistication measures including word frequency, frequency range, bigram and trigram frequency, academic language, and psycholinguistic word information. For example, TAALES calculates word range features that measure how many texts a word occurs in the SUBTLEXus corpus (Brysbaert & New, 2009), which is a 51-million-word corpus of subtitles from films and television series from the United States. TAALES can also calculate the imageability of words (i.e., how imageable a given word is as rated by humans) using MRC Psycholinguistic Database (Coltheart, 1981) and the concreteness of a word (i.e., how concrete a word is as rated by humans) using Brysbaert concreteness norms (Brysbaert et al., 2014). TAALES also includes age of exposure (AOE; Kuperman et al., 2012) norms based on a computational model of lexical acquisition derived from latent topic probability distributions (Dascalu et al., 2016).

TAALES also measures the number of phonographic and orthographic neighbors that a word has using norms reported by the English Lexicon Project (Balota et al., 2007). The ELP measures the number of neighbors a word has by calculating the number of words that differ in only one orthographic letter and one phoneme (e.g., *stone* and *stove*), including homophones or by examining the logged frequency of a word’s 20 closest neighbors, as determined by the orthographic Levenshtein distance, which is based on the hyperspace analogue to language (HAL) corpus (Lund & Burgess, 1996). TAALES can also measure the lexical and semantic associations a word has. For instance, TAALES uses semantic distinctiveness (SEM D; Hoffman et al., 2013) to measure the variety of semantic contexts in which a given word appears in. TAALES also reports on the University of South Florida (USF) free association norms (Nelson et al., 1998), which reports the number of stimuli words that a speaker associates with the target word. As well, TAALES includes Latent Semantic Analysis (LSA, Landauer et al., 1998) cosine similarity scores for words by reporting the average LSA cosine value between a given word and the top three words that have the strongest cosines with that word (i.e., the top three strongest related words). Words with stronger associations will report larger cosines with related words than words with weaker associations. Lastly, TAALES includes psycholinguistic measures of word processing including word naming (WN) reaction times and lexical decision (LD) accuracy for given words (Balota et al., 2007). These features measure the length of time it takes to decide that a word is a real word in English (versus a non-word) and the average accuracy of these decisions for each word.

### TAALED.

The Tool for the Automatic Analysis of LExical Diversity (TAALED, version 1.4.1; Kyle et al., 2020) calculates indices of lexical diversity using part-of-speech tagging and lemmatization. Measures of lexical diversity are related to lexico-semantics in that they capture the range and diversity of vocabulary in text. As such, they can assess the likelihood of a text incorporating a larger vocabulary and/or greater variation in vocabulary, which makes a text more difficult to read (Collins-Thompson, 2014). TAALED reports the number of types (i.e., unique words in a text) and tokens (i.e., number of words in a text) as well as classic measures of type-token ratio (TTR). TAALED also reports more advanced measures of lexical diversity such as the Measure of Text Length and Diversity (MTLD, McCarthy & Jarvis, 2010).

### TAACO.

The Tool for the Automatic Analysis of COhesion (TAACO, version 2.0.4; Crossley et al., 2016, Crossley et al., 2019a, b) calculates over 150 measures of local cohesion and global cohesion. These features are related to the discourse structure of texts. Texts are not randomly organized but rather have a structure that demonstrates relationships between elements in text such that some elements are dependent on others. Local cohesion refers to relationships that connect text elements that are spatially close (i.e., sentences) whereas global cohesion connects text elements that are farther apart (i.e., paragraphs or book chapters, Crossley et al., 2017a, b). Cohesion relationships help develop the coherence of text and lower text cohesion can affect the readability of text (Collins-Thompson, 2014).

In terms of local cohesion, TAACO reports on a wide range of connectives used to link sentences including temporal connectives (e.g., as *a consequence of, after,* and *again)* as well as lexical and semantic overlap between adjacent sentences (e.g., *Adjacent argument overlap*, which measures the number of noun and pronoun lemma types shared across sentences). In terms of global cohesion, TAACO measures lexical and semantic overlap among paragraphs.

### TAASSC.

The tool for The Automatic Analysis of Syntactic Sophistication and Complexity (TAASSC, version 1.3.8; Kyle, 2016) reports on clausal and phrasal indices of syntactic complexity as well as usage-based indices of syntactic sophistication. More complex syntactic features have been shown to slow text processing and measures of syntax taken from deep parses of sentence structures can reliably measure syntactic complexity (Collins-Thompson, 2014).

For example, TAASSC measures clausal complexity by calculating the number of nominal subjects per clause and phrasal complexity is measured by calculating the number of dependents per nominal subject. TAASSC also includes usage-based indices based on reference criteria reported by the Corpus of Contemporary American English (COCA; Davies, 2008), which includes sub-corpora specific to academic, fiction, magazine, and newspaper writing. TAASSC calculates attested items in a text, which measures the percentage of attested verb argument constructions found in COCA. TAASSC also calculates collostructional associations, which measure the strength of attraction that a word exhibits to a verb argument construction.

### SEANCE.

The tools for Sentiment Analysis and Cognition Engine (SEANCE, version 1.2.0; Crossley et al., 2017a, b) measures language features related to higher-level semantics and pragmatics. Higher-level semantics interacts with text readability because shared domain knowledge helps ease text comprehension. Pragmatic features help to capture subjective aspects of meaning that may help maintain reading engagement and motivation (Collins-Thompson, 2014). In terms of higher-level semantics, SEANCE reports on features related to cognition and social order. In terms of pragmatics, SEANCE reports measure of sentiment. For both, SEANCE includes incidence counts based on negation and part-of-speech (POS) tags. These counts are taken from different databases such as General Inquirer (GI; Stone et al., 1966), Lasswell (Lasswell & Namenwirth, 1969), and EmoLex (Mohammad & Turney, 2010, 2013). As an example, the GI database includes word lists that capture higher-level semantics related to achievement of goals, apart from whether the action may continue (i.e., complete words) and lists referring to identifiable and standardized individual human behavior patterns (i.e., roles), state verbs that indicate mental or emotional states (e.g., *believe, condone, fear, love, need, want*), and words for socially defined interpersonal processes (i.e., social relations). From Lasswell, SEANCE reports indices related to a general space-time category (higher-level semantics) and anticipation (sentiment). From EmoLex, SEANCE reports on emotional valence (i.e., the presence of positive or negative words).

### Statistical analyses

To assess associations between the CLEAR ease of readability scores for the full model and the data split and the readability formulas, we used the corrplot package (Wei & Simko, 2021). To develop a linear model of text readability found in the CLEAR scores, we initially started with 799 NLP features derived from SALAT. These NLP features related to lexical-semantics, syntax, discourse, higher level semantics, and pragmatics. For the linear model, we first removed features that had high zero counts (above 20%). This removed 398 features, leaving use with 401 NLP features. We then calculated bivariate Pearson correlations using the cor.test() function to identify highly collinear features. If two or more variables correlated at *r* > .699, the NLP variable(s) with the lowest correlation with the ease of readability score was removed and the variable with the higher correlation was retained. We also only retained variables that demonstrated at least a small relationship with the ease of readability scores (*r* > .099, Cohen, 1992). This removed 294 features, leaving us with a final feature set of 107 indices.

We used the CARET package (Kuhn, 2008) in R to develop linear models using the final 107 features. Model training and evaluation were performed using a stepwise tenfold cross-validation. For the stepwise process, we used the leapSeq function in Leaps (Kuhn et al., 2020). In the tenfold cross-validation procedure, the entire corpus was randomly divided into ten roughly equivalent sets and nine of these sets were used as a training set and one set was left out as a test set.^1^ The model from the training set was then applied to the left-out test set. This happened ten times such that each set was used as the test set once. Estimates of accuracy are reported using average summary statistics across the ten test sets including root mean squared error (RMSE), mean absolute error (MAE) between the observed and modeled human scores, and the amount of variance explained by the developed model (*R2*). The model reported was then entered into a standard linear model to retrieve an *F* value and *t* values for each included variable, and relative importance metrics for the included variables. For the linear models, we used the lm() function (R core team, 2020). The relative importance of the indices in each model was calculated using the calc.relimp() function in the relaimpo package (Grömping, 2006). Specifically, the metric lmg (Lindeman et al., 1980), which takes into account both the direct relationship between the independent and dependent variable (i.e., the bivariate correlation) and the indirect relationship between the independent and dependent variable (i.e., the amount of variance explained when included in a multivariate model), was used. The R code used in this study is available at https://github.com/scrosseye/CLEAR-Corpus.

## Results

### Correlations with existing readability formulas

We examined correlations between the ease of readability scores reported for the full model and for the two data split and both the classic and newer readability formulas calculated by ARTE. Correlations for this analysis are reported in Fig. 4. The results indicate strong overlap between all selected readability formulas and the readability ease scores for the full dataset reported by the Bradley–Terry model. The strongest correlations were reported for CAREC while the weakest correlations were reported for the Automated Readability Index. While strong, the correlations indicate that the readability formulas only predict around 23–34% of the variance in the reading ease scores. Lower, but moderate correlations, were reported between the data splits and the readability formulas. The correlations followed the same pattern as found in the full Bradley–Terry model. It should also be noted that many of the readability formulas were highly multicollinear including Flesch Reading Ease, Flesch–Kincaid Grade Level, the Automated Readability Index, and the New Dale–Chall Readability Formula.

## Linear model of readability

When the final 107 variables were entered into a tenfold cross-validated linear model, the number of variables that performed the best in explaining the reading ease score, after controlling for suppression effects, was 28. The linear model reported RMSE = .726, MAE = .575, *r* = .712, *R2* = .507, *F* (28, 4695) = 176.90, *p* < .001 (see model parameters summarized in Table 3). The relative importance metrics indicate that the strongest predictors of reading ease were related to ease of word decoding leading to greater readability (e.g., words that were more common, concrete, and attested along with words that were learned earlier and had more associations). There were also a number of syntactic features that were predictive, indicating that more complex syntactic structures, especially at the noun phrase level, led to lower readability. In addition, a number of sentiment and cognition terms were predictive of readability. These terms generally indicated that features that were likely associated with literary texts (i.e., more terms related to time and space, social relations and roles, and goal achievement) were related to greater reading ease as we were terms related to positive affect. Lastly, cohesion indices were also significant predictors with more instances of local cohesion (e.g., overlap between sentences and temporal connectives) associated with reading ease.

**Table 3:** Linear model to predict reading ease score

Variable	Tool	Relative Importance	Estimate	SE	*t*	*p*
(Intercept)			– 0.958	0.011	– 91.016	< 0.001
Word range: SUBTLEXus, CW logged	TAALES	0.169	0.345	0.024	14.106	< 0.001
Attested constructions: COCA fiction, lemmas	TAASSC	0.080	0.085	0.017	4.892	< 0.001
Word naming speed: standard deviation, CW	TAALES	0.075	– 0.026	0.015	– 1.742	< 0.100
Word concreteness: Brysbaert, CW	TAALES	0.072	0.042	0.018	2.384	< 0.050
Word age of exposure: LDA	TAALES	0.071	– 0.050	0.015	– 3.335	< 0.001
Lexical decision accuracy	TAALES	0.051	0.107	0.012	8.812	< 0.001
Positive words	SEANCE	0.048	0.033	0.015	2.253	< 0.050
Nominal subject per clause	TAASSC	0.040	0.034	0.013	2.685	< 0.001
Word imageability: MRC	TAALES	0.036	0.122	0.021	5.909	< 0.001
Number of word types	TAALED	0.033	– 0.117	0.012	– 9.726	< 0.001
Semantic variability: D	TAALES	0.031	– 0.168	0.017	– 9.975	< 0.001
Word association strength: LSA average top 3 cosine	TAALES	0.029	0.056	0.014	3.962	< 0.001
Argument overlap: Adjacent sentences	TAACO	0.028	0.040	0.012	3.315	< 0.001
Attested constructions: COCA news	TAASSC	0.027	0.026	0.015	1.766	< 0.100
Word range: SUBTLEXus, FW	TAALES	0.024	0.051	0.013	4.084	< 0.001
Words related to social relations	SEANCE	0.022	0.040	0.012	3.396	< 0.001
State words	SEANCE	0.020	0.069	0.014	4.826	< 0.001
Collexeme ratio: COCA fiction	TAASSC	0.019	0.027	0.013	2.183	< 0.050
Word association strength: USF, FW	TAALES	0.018	0.031	0.012	2.676	< 0.010
Words related to goals	SEANCE	0.018	0.044	0.011	3.841	< 0.001
Number of noun phrase dependents	TAALES	0.015	– 0.025	0.011	– 2.255	< 0.050
Phonographic neighbors	TAALES	0.014	– 0.036	0.014	– 2.664	< 0.001
Orthographic neighbors	TAALES	0.013	– 0.024	0.014	– 1.769	< 0.100
Words related to space and time	SEANCE	0.011	0.058	0.013	4.457	< 0.001
Temporal connectives	TAACO	0.010	0.022	0.012	1.884	< 0.100
Words related to roles	SEANCE	0.010	0.021	0.012	1.74	< 0.100
Collexeme ratio: COCA, types	TAASSC	0.009	0.029	0.012	2.392	< 0.050
Words related to anticipation	SEANCE	0.005	0.023	0.012	1.993	< 0.050

*CW* content words, *FW* function words

## Discussion

The purpose of the second study was to examine the reliability of the human scores of text readability through text analyses. The underlying premise was to examine whether readability formulas and text features known to be related to text comprehension were associated with the CLEAR ease of readability scores. To do this, we conducted two studies. The first examined overlap between existing readability formulas and the CLEAR ease of readability scores with the presumption that strong associations would be reported between them. Strong associations would indicate that the text features that inform existing readability formulas are also predictive of the human ease of readability scores. However, such analyses do not provide us with information about what individual features known to be related to text comprehension found in the excerpts are predictive of human judgements of text readability (i.e., which features in the excerpts may have influenced the human ratings). Thus, we conducted a second analysis in which we developed a readability formula to predict the readability scores and better understand links between individual features in the texts and their strength in predicting text readability.

Our examination of existing readability formulas indicated that both traditional and newly developed readability formulas showed strong relationships (*r* > .500, Cohen, 1992) with the CLEAR scores (except for the ARI scores). The strongest correlations were reported with CAREC (*r* = .580), the most advanced readability formula we sampled. In total, the results indicate that existing readability formulas are predictive of the CLEAR scores, but only explain about 23–34% of the variance (as derived from *R2* scores). Additionally, the readability formulas do not provide information on the language features in the text that may be influencing the raters scores of text understanding.

Thus, we conducted a follow-up analysis wherein we used open-sourced NLP tools to extract text information from the excerpts related to lexical sophistication, lexical diversity, syntactic complexity, cohesion, sentiment, and cognition. We developed a readability model that included 28 text features and explained over 50% of the variance in the CLEAR scores. As hypothesized, the strongest predictors were related to word decoding and indicated that excerpts with less sophisticated words were easier to understand. Additionally, excerpts that had syntactic features that were easier to parse led to texts that were easier to understand. There were also a number of cognition features related to narrativity (i.e., greater use of time and space and social relations) that were positively predictive of text ease of readability. From a sentiment perspective, excerpts that were more positive were easier to understand than negative excerpts, and from a cohesion perspective, excerpts that had greater local cohesion led to easier to comprehension. Together, these features indicate that text features known to influence text processing and comprehension were strong indicators of CLEAR scores providing support that these features influenced raters’ judgments and providing reliability for the scores themselves.

## Conclusions

We introduce the CommonLit Ease of Readability (CLEAR) corpus and the CLEAR scores and assess their reliability using a variety of metrics. The CLEAR corpus provides unique readability scores for ~ 5000 excerpts leveled for 3rd–12th grade readers along with information about the excerpts’ year of publishing, genre, and other meta-data. The CLEAR corpus will provide reading researchers and researchers interested in discourse processing with a resource from which to develop and test readability metrics and to model text readability. Moreover, it provides a number of improvements over previous readability corpora.

First, the CLEAR corpus is much larger than any available corpora that provide readability criterion based on human judgments. While there are large corpora that provide leveled texts (e.g., The Newsela corpus), these corpora only provide indications of reading ability based on discrete levels of simplification (i.e., beginning texts as compared to intermediate texts). The corpora do not provide readability criterion for individual texts. Individual reading criteria, like that reported in by the CLEAR scores, allows for the development of linear models of text readability. While there are other corpora that have reading criteria for individual texts, the corpora are much smaller (*N* = ~ 20–600 texts), and they do not contain the breadth of texts found in the CLEAR corpus. The size of the CLEAR corpus ensures wide sampling and variance such that readability formulas derived from the corpus should be strongly generalizable to new excerpts.

The breadth of excerpts found in the CLEAR corpus is an additional strength. The corpus was curated from the texts available on the CommonLit website, all of which have been specially leveled for a particular grade level. The CommonLit excerpts were supplemented by hand selected excerpts taken from Project Gutenberg, Wikipedia, and dozens of other open digital libraries. The text excerpts were published over a wide range of years (1791–2020) and are representative of two genres commonly found in the K–12 classroom: informational and literary genres. The texts were read by experts to ensure they matched excerpts used in the K–12 classroom and checked for appropriateness using MPAA ratings. All texts were hand edited, so that grammatical, syntactic, and spelling errors were limited.

A final strength is the reading criteria developed for the CLEAR Corpus (i.e., the CLEAR scores). Previous studies have developed reading criteria based on cloze tests or multiple-choice tests, both of which may not measure text comprehension accurately (Magliano et al., 2007). Additionally, while many readability formulas are marketed for K–12 students, their readability criteria are based on a different population of readers. The best example of this is Flesch–Kincaid Grade Level, which was developed using reading tests administered to adult sailors. We bypass these concerns, to a degree, by collecting judgments from schoolteachers about how difficult the excerpts would be for their students to read. This provides greater face validity for our readability criteria, which should translate into greater predictive power for readability formulas developed on the CLEAR corpus. However, caution is warranted in that human judgments of readability are subjective and likely contain error as well.

Lastly, while the purpose of the CLEAR corpus is for the development of readability formulas, the corpus includes metadata that will allow for interesting and important sub-analyses. These analyses would include investigations into readability differences based on year of publication, genre, author, and standard errors, among many others. The sub-analyses afforded by the CLEAR corpus will allow greater understandings of how variables beyond just the language features in the excerpts influence text readability.

## Footnotes

1 K-fold cross-validation without nested loops may lead to overfitted models that do not generalize outside of the dataset on which they are trained. However, since our interest is understanding links between the linguistic features and text readability and not generalizability, we did not use nested cross-validation.
